# Multiple testing in orthopedic literature: a common problem?

**DOI:** 10.1186/1756-0500-6-374

**Published:** 2013-09-21

**Authors:** Monique MJ Walenkamp, Kit CB Roes, Mohit Bhandari, J Carel Goslings, Niels WL Schep

**Affiliations:** 1 Trauma Unit, Department of Surgery, Academic Medical Center, University of Amsterdam, P.O. Box 22660, Amsterdam 1100 DD, The Netherlands; 2 Julius Center for Health Sciences and Primary Care, University Medical Center, Utrecht, Huispost Str. 6.131, P.O. BOX 85500, Utrecht, GA 3508, The Netherlands; 3 Department of Orthopedics, Hamilton Health Sciences-General Hospital, 237 Barton Street, 5 North, Hamilton, ON L8L 2X2, Canada

**Keywords:** Type one error, Multiple testing, Bonferroni, Orthopedic literature, Family wise error rate

## Abstract

**Background:**

Performing multiple tests in primary research is a frequent subject of discussion. This discussion originates from the fact that when multiple tests are performed, it becomes more likely to reject one of the null hypotheses, conditional on that these hypotheses are true and thus commit a type one error. Several correction methods for multiple testing are available. The primary aim of this study was to assess the quantity of articles published in two highly esteemed orthopedic journals in which multiple testing was performed. The secondary aims were to determine in which percentage of these studies a correction was performed and to assess the risk of committing a type one error if no correction was applied.

**Methods:**

The 2010 annals of two orthopedic journals (A and B) were systematically hand searched by two independent investigators. All articles on original research in which statistics were applied were considered. Eligible publications were reviewed for the use of multiple testing with respect to predetermined criteria.

**Results:**

A total of 763 titles were screened and 127 articles were identified and included in the analysis. A median of 15 statistical inference results were reported per publication in both journal A and B. Correction for multiple testing was performed in 15% of the articles published in journal A and in 6% from journal B. The estimated median risk of obtaining at least one significant result for uncorrected studies was calculated to be 54% for both journals.

**Conclusion:**

This study shows that the risk of false significant findings is considerable and that correcting for multiple testing is only performed in a small percentage of all articles published in the orthopedic literature reviewed.

## Background

Hypotheses testing, or in a narrower sense, assessing differences between groups of patients is frequently the primary aim of (clinical) studies. Traditionally, the results of these tests are translated into p-values and declared either significant or non-significant. An accepted mathematical definition of the p-value is that it represents the probability of the observed result, or more extreme results, if the null hypothesis were true [1]. Another frequently used term is the *type 1 error,* which is the rejection of a correct null hypothesis. The probability of committing such an error is often referred to as the *level of significance*[2]. An arbitrary threshold value for this level of significance, denoted by the Greek letter alpha, is set in advance. By convention most studies employ an alpha of 0.05. Accordingly, an obtained p-value of less than 0.05 (thus if p < alpha) is defined as a statistically “significant” association. One could state that a significant difference is thus an observation which is unlikely (< 5%) to have occurred by chance alone.

In clinical studies, researchers may wish to compare groups on several different parameters and therefore perform multiple statistical tests. However, problems arise if a set of statistical inferences are considered simultaneously. Or put differently: when groups of patients are compared on multiple variables using an equal number of statistical tests, and each significant difference is declared as such. When multiple tests are performed, it becomes more likely to reject at least one null hypothesis, conditional on that the hypotheses are true, and thus commit a type one error. Or clinically: attributing the difference found to the intervention under study when chance is the most likely explanation.

Multiple testing especially becomes an issue when no primary outcome is predefined, since this poses a risk for so-called ‘data dredging’, or ‘p-value hunting’ by the investigators. A classic example is comparing two groups of patients on the results of many different types of blood tests. As the number of tests increases, so does the probability of finding an abnormal result, while in fact there is no abnormality present.

Moreover, univariate multiple testing like this does not take into account any correlation which might be present between variables. For example: comparing a group of patients suffering from osteoporosis to a group of healthy individuals on ten different parameters. Some of these variables such as weight, smoking or dietary patterns are correlated. Performing ten separate tests ignores these correlations. Thus an observed difference on one variable might be explained by other differences between groups [3].

A currently well-accepted statistical approach is to control the Family Wise Error Rate: the probability of falsely rejecting a null hypothesis among a pre-defined family of hypotheses. In a simplified situation of a family of *n* hypotheses that can be tested with statistically independent tests, each at level α, this error rate would be: α = 1 - (1 – α (per comparison))^n^[4]. If for instance a set of twenty independent variables were tested, all at a 0.05 level of significance, the Family Wise Error Rate would be 1 - (1–0.05)^20^ = 0.64. Assuming independence between associations, this figure indicates that there is a 64 percent chance that at least one variable shows a significant difference while this difference in reality is nonexistent. Several elegant correction methods for multiple testing exist such as Bonferroni, Šidák, Benjamini & Hochberg and Holm’s [4-6], each suited for specific types of multiple testing.

The primary aim of this study was to assess the number of articles published in two highly esteemed orthopedic journals in which multiple testing was performed. The secondary aims were to determine in which percentage of these studies a correction was performed and to assess the risk of committing a type one error if no correction was applied.

## Methods

### Eligibility, information sources, search and study selection

The 2010 annals of two orthopedic journals: the Journal of Bone and Joint Surgery American Edition and Journal of Bone and Joint Surgery British Edition (hereinafter referred to as A and B) were systematically hand searched by two independent investigators. All titles were screened and publications reporting original or primary research were identified. If titles did not provide sufficient information, abstracts were examined. Case studies, meta-analyses, reviews, comments and current (management) views were excluded. Eligibility of original research articles was assessed based on full text. Any type of study in which the use of statistics was mentioned in the methods section was considered. These articles were thoroughly reviewed to identify cases of multiple testing. Multiple testing was predefined as five or more p-values obtained from comparing two or more groups on a set of variables, using separate statistical tests. Articles were thoroughly reviewed for tables or graphs in which five or more p-values were listed or significance between groups was marked with a sign. Publications were included if (1) five or more p-values were reported *and* if (2) the reported p-values were obtained from comparing two or more groups on a set of variables *and* if (3) separate statistical tests for each variable were used. Exclusion criteria were: (1) p-values obtained from baseline tables after randomization and (2) p-values originating from fitting models (linear, logistic, mixed). The above-mentioned criteria were assessed by carefully reviewing the method section and table legends. Discrepancies were solved by a consensus between the two investigators (MMJW, NWLS).

### Data extraction and items

Data were extracted by each investigator independently using a data collection form. This form contained the following items: type of study, sample size, type of statistical tests performed, number of p-values presented (obtained from comparing two or more groups on a set of variables using separate statistical tests), and number of these p-values < 0.05 or denoted as representing a significant association by the authors. In addition to this, the reviewers established whether a method of correction was mentioned, the primary outcome was stated in the introduction section, and whether a statistician or epidemiologist was listed as one of the authors.

### Statistical methods

Data were analyzed according to journal; A or B. Values are presented as median with interquartile range for continuous and percentages for dichotomous data. The theoretical risk of finding a significant statistical result, assuming the null hypotheses are true (type one error) and assuming independence, was calculated using the formula explained above (Family Wise Error Rate: α = 1 - (1 - α (per comparison))^n^). This was done for uncorrected studies only. A Fisher Exact test was performed to assess whether the presence of an epidemiologist or statistician in the research group was associated to correction for multiple testing.

## Results

**Figure 1:**
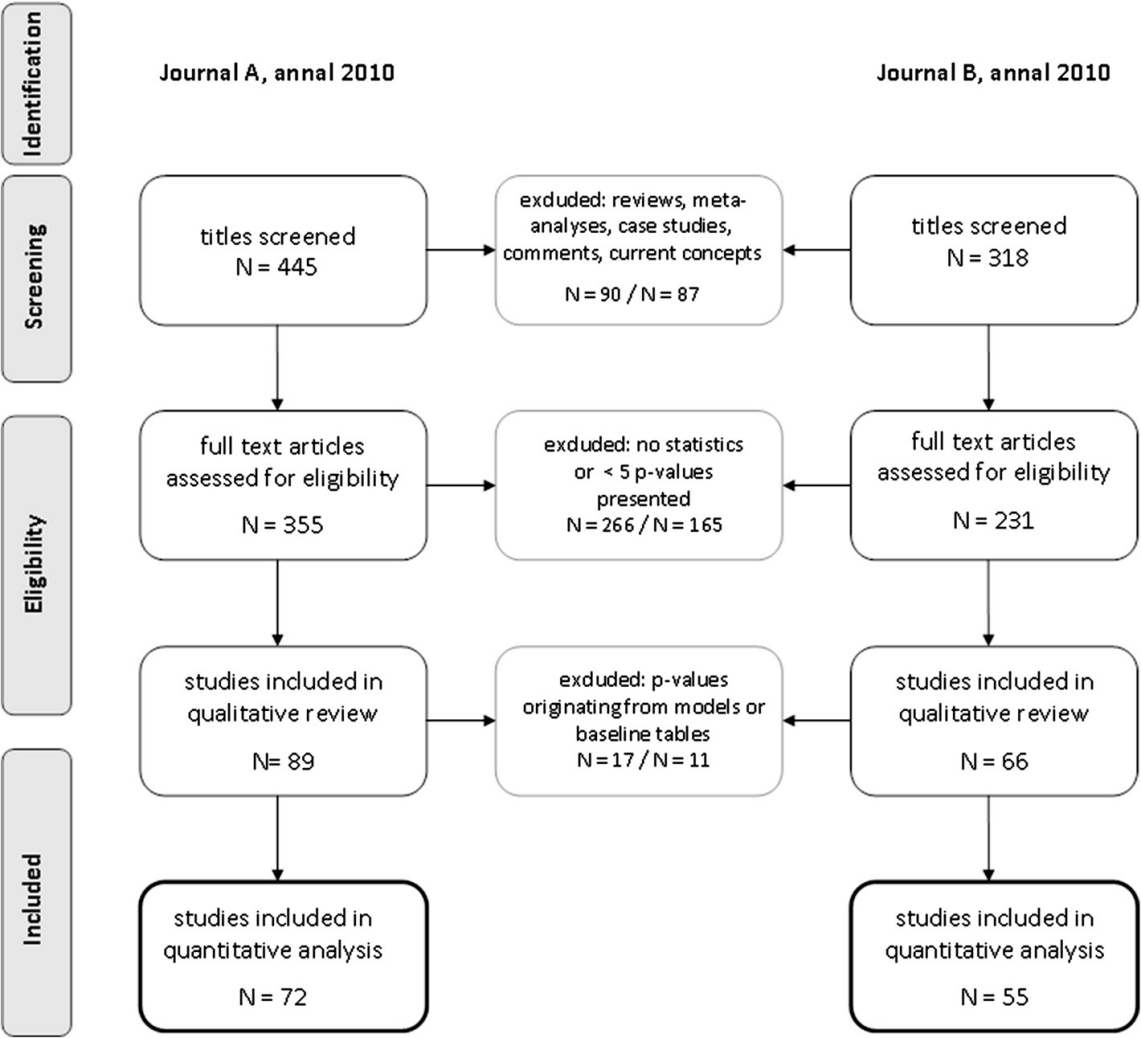
Flowchart; flowchart indicating the results.

A total of 763 titles were screened; 445 of journal A and 318 of journal B. Of these, 355 and 231 articles were assessed for eligibility. After carefully reviewing all articles with regards to the predetermined criteria, 72 publications from journal A and 55 from journal B were included in the analysis (see Figure 1). The characteristics of the studies reviewed are listed in Table 1. Cohort studies most commonly employed multiple tests, followed by randomized controlled trials. The primary outcome was stated in 16% of the articles published in journal A and 26% in B. In 9% an epidemiologist or statistician was listed as one of the authors.

**Table 1:** Characteristics of studies included in the analysis

		**Journal A (N = 72) number of studies (%)**	**Journal B (N = 55) number of studies (%)**
Study type	RCT^$^	12 (16.7)	10 (18.2)
	Cohort	42 (58.3)	32 (58.2)
	Case Control	5 (6.9)	5 (9.1)
	Cross Sectional	1 (1.4)	2 (3.6)
	Cadaver	9 (12.5)	0
	Animal	1 (1.4)	1 (1.8)
	Other	2 (2.8)	5 (9.1)
Primary outcome stated		19 (26)	9 (16.4)
Statistician/epidemiologist part of research group	*		5 (9.1)
Corrected for multiple testing		11 (15)	3 (5.5)
Problem mentioned, not corrected		4 (6)	0

^$^Randomized controlled trial.

*Data not available: background of authors was not mentioned in articles published in this journal.

In the articles published in journal A, a total of 1531 p-values originating from multiple testing was listed. Of these, 37% was smaller than 0.05 (Table 2). In journal B, 1046 p-values were presented of which 36% was smaller than 0.05. A median of 15 statistical inference results were reported per publication in both journal A and B.

**Table 2:** Statistical results of studies included in the analysis

	**Journal A (N = 72)**	**Journal B (N = 55)**
Total number of p-values presented	2555	1783
Number of p-values originating from multiple testing	1531	1046
Number of p-values ≤ = 0.05	563 (37%)	377 (36%)
Median number of p-values per publication^#^	15 (8–33)	15 (8–21)
Median risk of obtaining at least one significant result^€^	54% (34–81)	54% (34–66)

^#^Values presented as median with interquartile range between brackets.

^€^Median risk and interquartile range expressed in percentages. Calculated for uncorrected studies using the formula for Family Wise Error Rate mentioned in the text, assuming independence and conditional on that the null hypotheses are true.

Correction for multiple testing was more often performed in journal A than B: 15% and 5.5% respectively (Table 1). In four articles from journal A the problem was mentioned, however not corrected for [7-10]. If corrected, Bonferroni was the method of choice in ten of the journal A articles and two from journal B [11-20], [21,22]. In one journal A publication, the authors mentioned a correction but did not describe the name or the method [23]. In one journal B study, the level of significance was set at α = 0.01 in order to interpret the results more conservatively [24]. The presence of an epidemiologist or statistician in the research group was not associated to the application of a correction for multiple testing.

The estimated median risk of obtaining at least one significant result for uncorrected studies was calculated to be 54% for both journals (Table 2).

## Discussion

This review shows that multiple testing is frequently performed in orthopedic literature and that correction methods are not widely applied. However, in order to fully appreciate these findings, several issues need to be addressed. First, performing multiple tests and the absence of corrections does not necessarily result in the arrival at false conclusions in literature. Nonetheless, false conclusions are not distinct and can only be identified by ascertaining their reproducibility in other studies. The validity of research findings in a broader sense has frequently been addressed [25], however, this subject is beyond the scope of this review. This study is limited by the fact that articles were not scanned for multiple tests mentioned in the text only, and neither were tables which only listed confidence intervals included, although this way of presenting results can also be considered to be multiple testing [26]. This restriction will most likely have resulted in an underestimation of the actual existing problem. P-values originating from fitting models (linear, logistic, mixed) were not taken into account since this type of analysis does not address the comparison of groups on a set of variables using independent statistical tests. The cut-off of five or more p-values was to some extent an arbitrary choice. Performing five statistical tests, assuming independence and conditional on that the null hypotheses are true, will result in an estimated 23% risk of committing a type one error (1 - (1–0.05)^5^ = 0.23). Since no standard threshold for multiple testing exists, the authors decided to draw the line at five. In view of the fact that on average 23 tests per article were reported in journal A and 21 per article in journal B, the threshold of five does not present a restriction that would seriously bias our conclusions.

Although multiple testing is frequently the subject of statistical recommendations, the issue is not undisputed [2,27]. Rothman for example, claims that adjustment is not required for multiple testing since the data observed are not just random numbers but actual observations on nature. Nature follows regular laws and adjusting for this data will lead to errors of interpretation [2,27]. Walker stated: “Should I discount an interesting finding because the investigator tested some hypotheses which I consider absurd?” [28].

Nevertheless, in 1992 already the editors of journal A addressed the issue of the frequent use of incorrect statistics in their journal [29]. They stated that, despite the controversy, multiple comparisons should be corrected or at least that the risk of error should be discussed. A possible method of correction for multiple testing is the Bonferroni correction. This method is based on the idea that if *n* hypotheses are tested on one set of data, the Family Wise Error Rate can be maintained by testing each individual hypothesis at a significance level of α/n, where n is the number of tests performed [4]. However, Bonferroni is often claimed to be too conservative and controls the probability of false positives only. The correction may result in increasing the probability of producing false negatives and therefore reduce the power. In contrast, in some situations the null hypothesis is wished to be retained and not rejected. In this case the Bonferroni correction is non-conservative since it favors the null hypothesis [30]. Numerous alternative correction methods to Bonferroni exist such as the Šidák, Benjamini & Hochberg and Holm’s correction [5,6]. These methods are applicable to specific types of multiple testing.

## Conclusions

Eighteen years after the editorial recommendations of journal A, the findings in this study indicate that the phenomenon of multiple testing is still common in orthopedic literature and that corrections are not widely applied. As was stated by the man who first proposed the term *significance*, R.A. Fisher: “*a scientific fact should be regarded as experimentally established only if a properly designed experiment rarely fails to give this level of significance*” [31]. Thus, the operational connotation of significance and p-values should always be viewed in light of their reproducibility in other studies.

Whilst the issue and the method of correcting for multiple testing remains disputed, we would like to recommend researchers to bear in mind that, as the number of tests increases, so does the probability of falsely rejecting the null hypothesis. This does not necessarily result in false conclusions; however, it would be advisable to mention some caution regarding the interpretation of the results (Table 3). Furthermore, in order to prevent the possible impression of data dredging, it might be sensible to clearly state the primary outcome measure(s) in the introduction.

**Table 3:** Key recommendations

1.	Predefine a primary outcome (and set your alpha based on this outcome)
2.	Predefine the secondary outcomes to avoid the appearance of “data dredging” or “p-value hunting”
3.	Mention some caution regarding the interpretation of that results yielded from multiple testing or perform a correction

## Competing interests

The authors declare that they have no competing interests.

## Authors’ contributions

All authors participated in the design and drafting of the manuscript. NWLS and MMJW carried out the systematic hand search. All authors have read and approved the final manuscript.
